# Circulating Cell-Free miR-25-3p Is Upregulated in Brazilian Patients with Prostate Cancer and Contributes to Tumor Aggressiveness

**DOI:** 10.3390/ncrna12050033

**Published:** 2026-08-30

**Authors:** Monyse Nobrega, Hellen Kuasne, Marilesia Ferreira de Souza, Mariana Bisarro dos Reis, Larissa Cristina Bastos de Oliveira, Paulo Emilio Fuganti, Morag Park, Ilce Mara de Syllos Cólus

**Affiliations:** 1 Department of General Biology, State University of Londrina, Londrina 86057-970, PR, Brazilcolus@uel.br (I.M.d.S.C.); 2 Rosalind and Morris Goodman Cancer Institute, McGill University, Montreal, QC H3A 1A3, Canada; 3 Molecular Oncology Research Center, Barretos Cancer Hospital, Barretos 14784-400, SP, Brazil; 4 Londrina Cancer Hospital, Londrina 86015-520, PR, Brazil; 5 Department of Biochemistry, McGill University, Montreal, QC H3G 1Y6, Canada; 6 Department of Medicine, McGill University, Montreal, QC H4A 3J1, Canada; 7 Department of Oncology, McGill University, Montreal, QC H4A 3T2, Canada

**Keywords:** prostate cancer, liquid biopsy, cell-free RNA, ctmiRNA, LNCaP, prognostic biomarker

## Abstract

**Background/Objectives:** Prostate cancer (PCa) has a high incidence worldwide, yet reliable prognostic biomarkers to distinguish indolent from aggressive forms are lacking. MicroRNAs (miRNAs), a class of small non-coding RNAs, are stable molecules that play an important role in cancer development and can be detected in circulation by non-invasive methods. This study aimed to examine the influence of the cell-free (cf) miRNAs levels on PCa risk and aggressiveness using plasma samples and cell models. **Methods:** Four overexpressed miRNAs in prostate cancer tissue were selected from The Cancer Genome Atlas database: miR-25-3p, miR-92a-1-5p, miR-92a-2-5p, and miR-148a-3p. Cf-miRNA levels were validated via quantitative reverse transcription polymerase chain reaction (RT-qPCR) in plasma samples from 80 PCa patients and 40 controls in a Brazilian cohort. Clinicopathological associations were analyzed to determine prognostic value. Subsequently, functional analyses were performed using transient transfection of miR-25-3p in the LNCaP cell line, assessing proliferation, migration, invasion, and target gene regulation. **Results:** Among the miRNAs analyzed, only miR-25-3p was upregulated in the plasma of PCa patients compared to controls (*p* = 0.013). Patients with International Society of Urological Pathology (ISUP) grade ≥2 presented higher expression levels of miR-25-3p (*p* = 0.020) compared to patients with ISUP grade 1. Overexpression of miR-25-3p significantly increased cell proliferation, colony formation, migration, and invasion capacity and modulated the expression of *BCL2L11*, *CDH1*, *CDKN1C*, *EHZ2*, and *TP53* genes. **Conclusions:** miR-25-3p had an oncogenic role in PCa, showing a potential marker to assess PCa development and progression using liquid biopsy.

## 1. Background

Prostate cancer (PCa) is the second most diagnosed cancer in men worldwide [1]. Despite its high incidence, the majority of cases follow a relatively indolent clinical course [2]. Although the 5-year relative survival rate is close to 100% for patients with early-stage tumors, it decreases to 30% for patients with late-stage disease [3]. A comprehensive analysis of autopsy-detected PCa has demonstrated that its prevalence increases with age, being identified in over 50% of men aged 90 years or older. Notably, the frequency of high-grade tumors approximately doubles with each additional decade of life, and more men die with PCa than from PCa [4].

Given the clinical challenge of distinguishing indolent from aggressive disease, more accurate biomarkers represent a critical unmet need. The most widely used biomarker, prostate-specific antigen (PSA), started to be utilized as a screening test in the late 1980s, with great promise due to its high sensitivity to the prostate and feasibility. Recently, advisory groups recommended against using PSA tests because of unsubstantiated outcomes [5,6,7]. Yet, due to the high heterogeneity of PCa, obtaining a representative tissue sample is challenging [4,8]. Among PCa diagnoses, approximately a third of the Gleason scores of PCa biopsies are inaccurate and underestimated, followed by metastasis within 5 years, which challenges clinical diagnosis and threatens patients’ lives [9]. These limitations underscore the need for alternative biomarker strategies that reduce reliance on invasive procedures, thereby improving the distinction between indolent and aggressive disease.

Several prognostic factors and risk-stratification approaches have been developed to distinguish indolent from biologically aggressive PCa. Among individual clinicopathological variables, ISUP grade group, clinical or pathological tumor stage, and serum PSA levels remain among the most established prognostic factors. Their combined assessment forms the basis of widely used risk-stratification tools, including the D’Amico risk classification, the Memorial Sloan Kettering Cancer Center (MSKCC) prostate cancer nomograms and the UCSF-CAPRA score, which were developed and validated using large patient cohorts [10,11,12]. Nevertheless, as the disease progresses, additional refinement of the prognosis may be required, utilizing PSA derivatives (such as PSA doubling time) and commercially available molecular assays, like Prolaris, Decipher, and Oncotype DX Genomic Prostate Score [13]. More recently, artificial intelligence-based models, such as ArteraAI, have also been investigated for prognostic and treatment-response stratification [14]. Despite these advances, currently available tools have limitations related to their applicability, cost, accessibility, and incremental prognostic value, supporting continued investigation of novel biomarkers capable of improving PCa risk stratification.

In this context, liquid biopsy (LB) has emerged as a promising minimally invasive approach, leveraging various biofluids for early detection, recurrence monitoring, therapeutic assessment, and prognostication [15]. Among circulating analytes, miRNAs have some inherent technical advantages over the others, which can be analyzed, such as stability in biofluids, relative abundance, and potential detectability even in early-stage disease [16,17]. In PCa specifically, numerous miRNAs are dysregulated in tumor tissue, and tissue-based classifiers demonstrate strong diagnostic and prognostic performance [18,19].

Although matched tissue–blood analyses could provide additional insight into the contribution of the tumor to circulating miRNAs levels, elevated cf-miRNA concentrations do not necessarily reflect proportionally increased expression within the tumor itself. Cf-miRNAs are dynamic signaling molecules whose systemic concentrations are governed by complex regulatory processes [20], for example, selective secretion into the bloodstream, including vesicular (small extra-cellular vesicles; microvesicles; apoptotic bodies) and/or non-vesicular (lipoprotein complexes; protein complexes; free/unbound) [21] and interactions with the tumor microenvironment such as hypoxia [22], rather than simply representing passive byproducts of tumor cell death. In addition, the marked spatial and molecular heterogeneity of prostate cancer should be considered. A focal biopsy sample may not fully capture the molecular landscape of the entire tumor.

In a previous study by our research group, an in silico discovery phase using The Cancer Genome Atlas (TCGA) database identified 49 differentially expressed miRNAs in prostate adenocarcinoma tissue compared to adjacent normal tissue. Subsequently, seven candidates were selected from these 49 and validated by RT-qPCR in plasma, which confirmed that miR-200c of the seven candidates was significantly associated with PCa diagnosis and prognosis [23]. However, the remaining candidates from the original set have not yet been investigated in a Brazilian population sample, leaving open the possibility that clinically informative miRNAs remain uncharacterized.

To address this gap, we expanded our analysis to include four additional miRNAs (hsa-miR-25-3p, hsa-miR-92a-1-5p, hsa-miR-92a-2-5p, and hsa-miR-148a-3p) that were significantly overexpressed in PCa tissue relative to matched normal tissue in the TCGA cohort. We recognized that miRNA overexpression in tumor tissue does not inherently translate to elevated levels in circulation. Furthermore, the cf-miRNAs currently validated in the literature derive predominantly from studies conducted in China, the United States, and Western Europe [24]. Given the unique genetic admixture and high PCa burden in Brazil, it is critical to investigate whether these signatures maintain their diagnostic and prognostic validity as circulating biomarkers across diverse genetic backgrounds. PCa-specific miRNAs are actively released into the circulation and can be detected through non-invasive approaches, we hypothesize that the selected miRNAs may serve as biomarkers in PCa using LB.

## 2. Results

### 2.1. Clinical Information

The total sample consisted of 120 men (80 PCa patients and 40 controls), of which 80.6% were European descendants and 19.4% were Afro-descendants. The mean age of patients was 70 (range 48−87 years), and that of controls was 64 (range 51–75 years). There were no statistically significant differences between PCa patients and controls for most of the characteristics studied, except for the family history of prostate cancer (*p* = 0.044). The PSA average in PCa patients was 38 ng/mL (range 1.51–933.9). Additionally, the detailed description of treatment, ISUP grading (International Society of Urological Pathology), tumor laterality, seminal vesicle invasion, perineural invasion, lymph node invasion, and bone metastases is described in Table 1.

### 2.2. Overexpression of miR-25-3p in the Plasma of PCa Patients Compared to Controls

**Figure 1:**
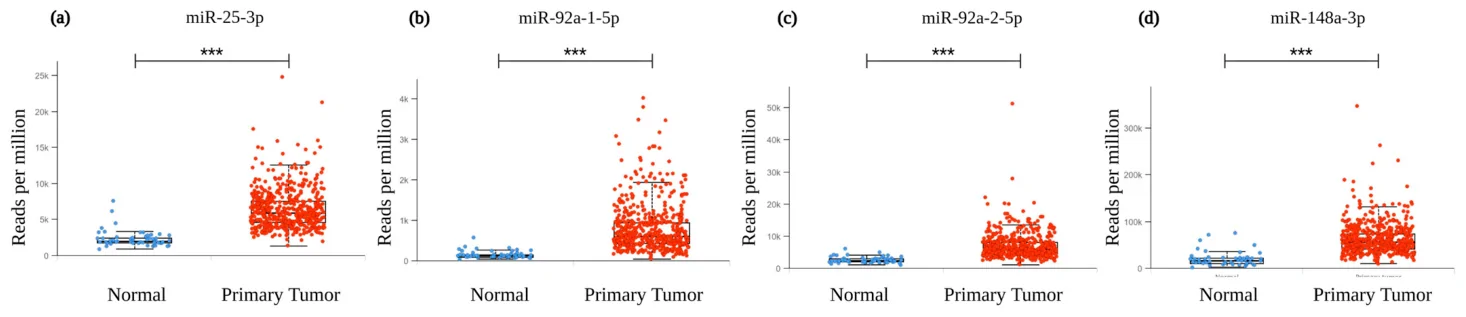
Expression level of the four selected miRNAs in prostate adenocarcinoma tumors and their adjacent normal tissue according to TCGA data downloaded from the UALCAN online tool. (**a**) miR-25-3p, (**b**) miR-92a-1-5p, (**c**) miR-92a-2-5p, (**d**) miR-148a-3p, *** *p* ≤ 0.001.

**Figure 2:**
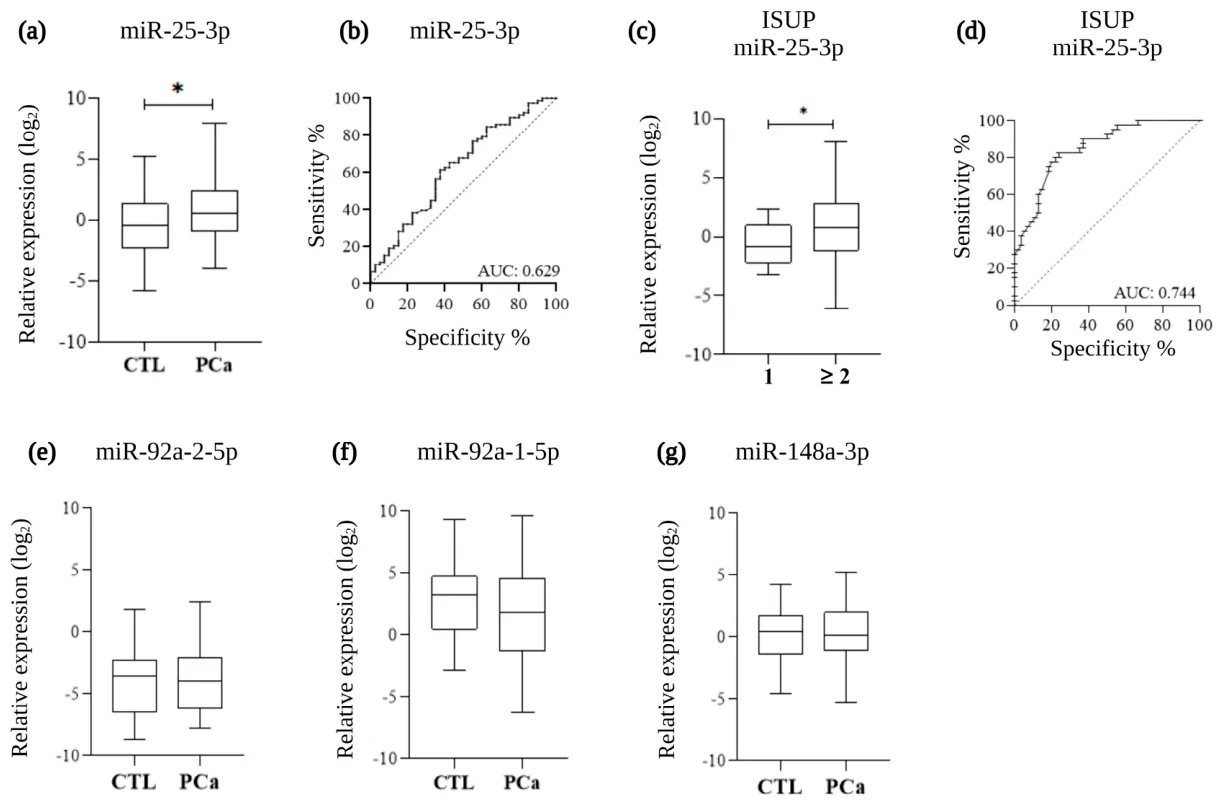
Circulating cell-free miRNA levels in PCa patients and controls. Differential expression of cell-free miRNAs in plasma samples of controls and PCa patients (**a**,**c**,**e**–**g**) and PCa patients according to the ISUP grading, (**c**) and their respective ROC curves, (**b**,**d**). PCa = prostate cancer; CTL = controls; ISUP grading = International Society of Urological Pathology; ROC = receiver operating characteristic; AUC = area under the curve; * *p* < 0.05.

The hsa-miR-25-3p, hsa-miR-92a-1-5p, hsa-miR-92a-2-5p and hsa-miR-148a-3p were confirmed as overexpressed in prostate adenocarcinoma tissue compared with the normal prostate dataset from TCGA (Figure 1a–d) (Supplementary Table S1). To evaluate their potential as circulating biomarkers using LB, we measured their expression levels in plasma samples from PCa patients and controls using RT-qPCR (Figure 2a–g). Among the miRNAs analyzed, only miR-25-3p showed statistically significant elevated levels in PCa patients compared to controls (*p* = 0.013; fold change = 2.15) (Figure 2a). ROC curve analysis was subsequently performed to assess its diagnostic performance, yielding a sensitivity of 61.5%, specificity of 62.5%, and an area under the curve (AUC) of 0.629 (95% CI: 0.520–0.737; *p* = 0.023) (Figure 2b). Neither the miR-92a-1-5p (Figure 2e), miR-92a-2-5p (Figure 2f), nor miR148a-3p (Figure 2g) showed statistical significance compared to controls.

While the expression levels of all miRNAs were evaluated in relation to various prognostic features, including tumor ISUP grade, laterality, seminal vesicle invasion, perineural invasion, lymph node metastasis, and bone metastasis, no statistically significant associations were observed, except for miR-25-3p. This miRNA consistently demonstrated a strong association with poor clinical outcomes. Specifically, patients with ISUP grade ≥2 exhibited miR-25-3p levels that were more than twofold higher than those observed in patients with ISUP grade 1 (*p* = 0.020; 3.49-fold increase) (Figure 2c). ROC curve analysis further demonstrated its ability to distinguish aggressive from indolent disease, with an AUC of 0.744 (95% CI: 0.520–0.833; *p* = 0.003), sensitivity of 80%, and specificity of 75.9% (Figure 2d).

### 2.3. Cell Proliferation, Colony Formation, Migration, and Invasion Are Enhanced by miR-25-3p Transfection

To test the functional role of miR-25-3p, we chose the prostate cancer cell line LNCaP. Given that LNCaP cells exhibit low endogenous levels of miR-25-3p, treatment with an anti-miR-25-3p resulted in no significant differences compared to the negative control (NC) in cell proliferation, colony formation, migration, or invasion (Supplementary Figure S1a–e). Therefore, this cell line is a suitable model for inducing the miR-25-3p abundance.

**Figure 3:**
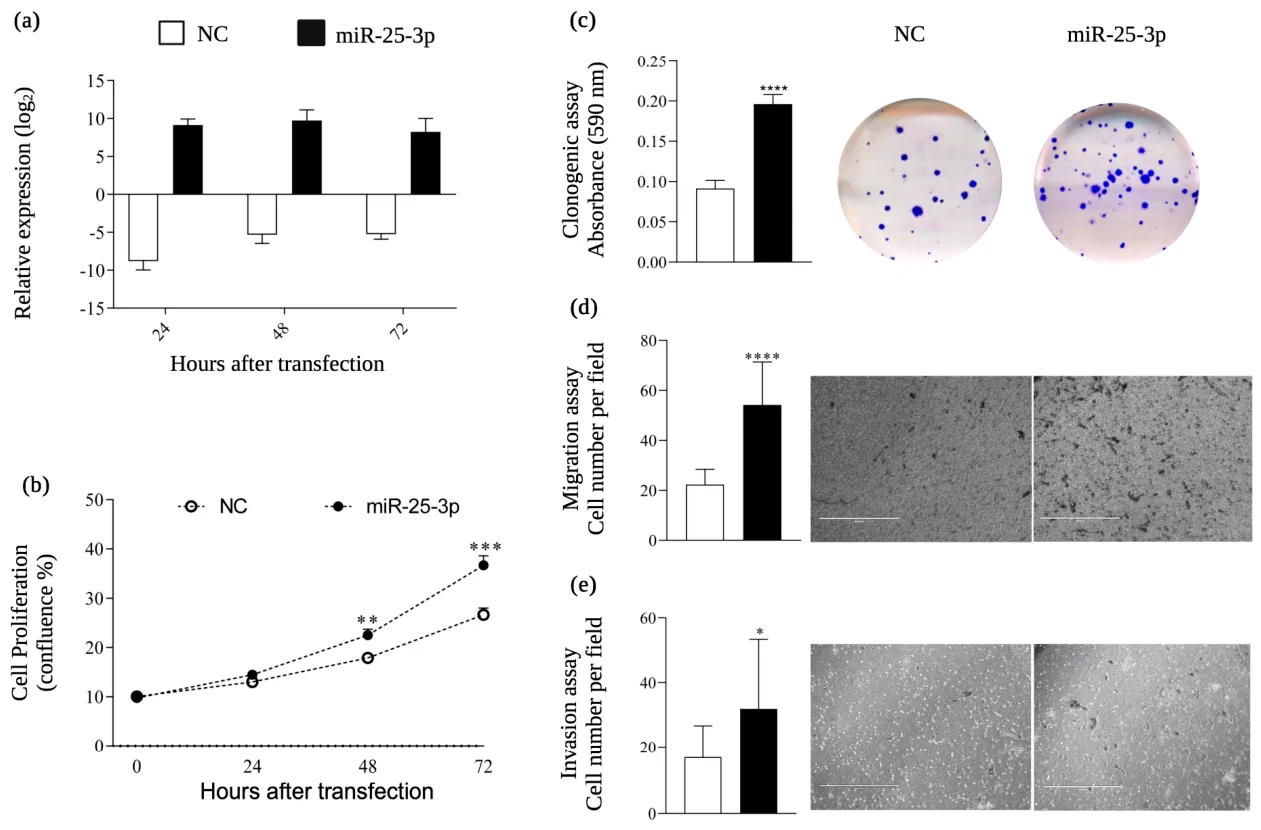
Functional validation of the miR-25-3p. (**a**) LNCaP cell lines transfected with miR-25-3p mimic compared to the NC to assess the transfection efficiency by RT-qPCR at different time points. (**b**) The proliferation was determined by IncuCyte^®^ live-cell analysis; (**c**) the clonogenic assay was used to assess the ability of cells to form colonies. Magnification: 1×; (**d**) the migratory capacity was checked using Boyden chamber assay. Magnification: 10×; (**e**) cellular invasion was measured using a transwell assay coated with Matrigel. Magnification: 10×. NC: negative control. * *p* < 0.05; ** *p* < 0.01; *** *p* < 0.001; **** *p* < 0.0001.

Consistently, compared to the NC, transfection with 10 nM of the miR-25-3p (mimic) resulted in increased miR-25-3p levels at all assessed time points (24, 48, and 72 h) (Figure 3a). We evaluated the proliferation rate of LNCaP cells after 24 h of transfection with NC and miR-25-3p. Cell proliferation images were captured every 8 h for 72 h after transfection using an Incucyte system. By applying a confluence mask to all images via the Incucyte S3 software to calculate cell surface area coverage, we observed that miR-25-3p transfection significantly enhanced cell proliferation at 48 and 72 h (Figure 3b; *p* = 0.007 and *p* = 0.0002, respectively). As shown in Figure 3c, after 14 days of transfection, miR-25-3p enhancement in LNCaP cells increased colony formation compared with NC cells. The relative colony-formation efficiency significantly increased the absorbance rate from 0.09 to 0.19 upon miR-25-3p overexpression. In addition, Boyden-chamber transwell migration and invasion assay revealed that migration (average NC: 22.30 vs. miR-25-3p: 54.17; *p* < 0.0001) (Figure 3d) and invasion (average NC: 16.83 vs. miR-25-3p: 31.89; *p* = 0.029) (Figure 3e) were enhanced in LNCaP cells after 48 h of transfection.

### 2.4. Elevated Levels of miR-25-3p Correlate with Downregulation of BCL2L11, CDH1, CDKN1C, EZH2, and TP53 in LNCaP Cells

**Figure 4:**
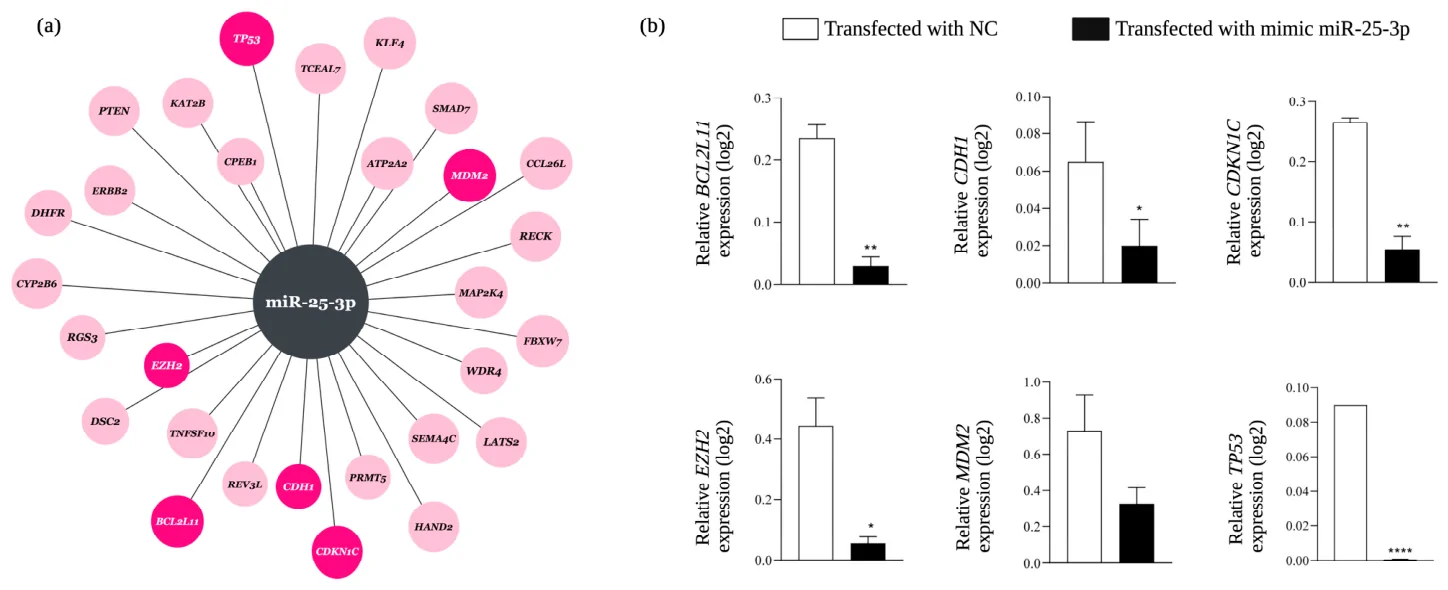
MiR-25-3p/mRNA target interaction. (**a**) Network of miR-25-3p and its predicted target genes, built from experimentally validated interactions (miRTarBase, via MIENTURNET); genes selected for functional validation are highlighted in dark pink; (**b**) gene expression of miRNA-25 targets using RT-qPCR after transfection of LNCaP cells with NC and miR-25-3p. Data are presented as mean ± SD. NC: negative control; * *p* < 0.05; ** *p* < 0.01; **** *p* < 0.0001.

Having observed that increased levels of miR-25-3p increased proliferation, clonogenic capacity, migration, and invasion in LNCaP cells (Figure 3), we sought to identify candidate target genes associated with this oncogenic phenotype. We used MIENTURNET/miRTarBase and identified 26 genes with strong interaction with miR-25-3p and potential relevance to PCa development and progression. From these 26 genes, we selected 6 genes associated with miR-25-3p: *TP53* and *MDM2*, directly implicated in PCa signaling; *BCL2L11, CDH1, CDKN1C*, and *EZH2* were additionally selected for their roles in epithelial–mesenchymal transition (*CDH1*), cell-cycle regulation (*CDKN1C*), epigenetic regulation of gene expression (*EZH2*) and apoptosis (*BCL2L11*) (Figure 4a; Supplementary Table S3).

The expression levels of *BCL2L11* (*p* = 0.007; FC = 0.08), *CDH1* (*p* = 0.013; FC = 0.31), *CDKN1C* (*p* = 0.005; FC = 0.21), *EZH2* (*p* = 0.028; FC = 0.12), and *TP53* (*p* < 0.0001; FC = 0.01) were significantly reduced in LNCaP cells upon miR-25-3p transfection. However, the *MDM2* expression was not affected by miR-25-3p overexpression in LNCaP cells (Figure 4b).

## 3. Discussion

The clinical adoption of LB represents a minimally invasive approach that enables molecular profiling from biofluids, offering several advantages over a single tissue biopsy core, including repeated sampling, real-time disease monitoring, and a more comprehensive representation of tumor heterogeneity [25].

The concept of “Liquid Biopsy” was coined in 2010 by Catherine Alix-Panabières and Klaus Pantel, when commercial methods for capturing circulating tumor cells (CTCs) became available [26]. LB encompasses a broader repertoire of circulating analytes, including ctDNA, circulating tumor cells (CTCs), extracellular vesicles (EVs), and circulating RNAs such as miRNAs, long non-coding RNAs, and messenger RNAs [26]. While each analyte class offers unique advantages for cancer detection and monitoring, all carry inherent limitations: for example, ctDNA presents low concentrations in some cancers and early-stages cancers [27], CTCs are rare and require a massive amount of blood [27], and EVs demand intricate purification methods [28]. Importantly, these analytes are not mutually exclusive; rather, their distinct biological and technical profiles suggest that integrating them into multimodal LB panels could overcome the individual limitations of each class and achieve diagnostic and prognostic performance beyond what any single biomarker can provide alone [18]. To date, FDA-approved LB tests have focused primarily on ctDNA-based platforms, including the Cobas EGFR Mutation Test v2 for non-small cell lung cancer, FoundationOne Liquid CDx, and Guardant360 CDx, which detect somatic mutations across multiple solid tumors, including PCa [25]. Due to the fast progress in the field, approval for new tests using LB with different analytes or multimodal LB panels is expected soon.

Among these analytes, miRNAs offer complementary strengths that address several of these shortcomings, including remarkable stability in circulation with half-lives around 16 h at room temperature and higher concentrations in early-stage disease [28,29].

Our study focused on miRNAs, which play pivotal roles in cancer progression, and they have been extensively investigated via LB as potential biomarkers for PCa, as previously reviewed by our group [24]. In the present study, we evaluated the plasma levels of miR-25-3p, miR-92a-1-5p, miR-92a-2-5p, and miR-148a-3p, all of which had previously been identified as overexpressed in PCa tissue. Interestingly, miR-25-3p was the only candidate that demonstrated significant upregulation in plasma, with expression levels approximately 2-fold higher in PCa patients than in non-cancer individuals.

We also observed higher levels of plasmatic miR-25-3p in patients with ISUP ≥ 2, suggesting its potential utility in distinguishing these patients from those with lower-grade disease. Patients with PCa Grade 1 can often be safely followed up through active surveillance (AS). In contrast, patients with grades equal to or above 2 can present poor prognoses, highlighting the potential of miR-25-3p as a prognostic biomarker. Accurate ISUP grade group classification demands considerable pathological expertise yet remains susceptible to meaningful interobserver variability even among specialists [27,30].

Our study did not evaluate miR-25-3p via longitudinal sampling to determine its prognostic value regarding overall survival; furthermore, miR-25-3p was not evaluated in combination with other established clinicopathological prognostic factors. Consequently, our findings do not support its immediate use as an independent prognostic biomarker in clinical practice. Nevertheless, the observed ability of miR-25-3p to discriminate between different ISUP grade groups, the single most important prognostic factor in prostate cancer, is of potential clinical interest, since tumor grade is strongly associated with PCa biological aggressiveness and clinical outcome. These findings suggest that miR-25-3p expression data could serve as an additional complementary tool to assist in ISUP classification, particularly in cases with inconclusive histopathological findings, and therefore support further evaluation of miR-25-3p in prospective longitudinal cohorts.

To our knowledge, this is the first study to evaluate cell-free miR-25-3p levels in a Brazilian cohort. Additionally, many other studies showed that miR-25-3p was upregulated in the serum or plasma in esophageal squamous cell carcinoma [31], non-small cell lung cancer [32], gastric cancer [33], colorectal cancer [34] and breast cancer [35]. Notably, our findings contrast with those of Cochetti et al., who reported, in an Italian cohort [36], that serum miR-25-3p was reduced in PCa patients compared to patients with benign prostatic hyperplasia (BPH). These discrepancies may be attributed to several methodological variations. First, plasma is often preferred over serum as it avoids the release of miRNAs during the coagulation process and generally provides greater miRNA diversity [37,38]. Therefore, plasma is typically considered a more reliable specimen for circulating miRNA analysis and is recommended by several studies [38,39,40,41]. Second, normalization strategies differed across studies. Whereas previous research utilized a combination of endogenous and exogenous controls, the absence of exogenous spike-in controls in our protocol represents a limitation that should be acknowledged [42]. Finally, the present study employed TaqMan probe-based assays, which provide greater sequence specificity than the SYBR Green-based detection methods [43].

In contrast to miR-25-3p, the expression levels of miR-92a-1-5p, miR-92a-2-5p, and miR-148a-3p did not differ significantly between patients with PCa and controls in our cohort, despite their reported overexpression in tumor tissue in the TCGA dataset. To the best of our knowledge, this is the first study to evaluate miR-92a-1-5p and miR-92a-2-5p in the LB setting. The literature regarding cf-miR-148a-3p in PCa remains highly inconsistent. Some studies have reported its upregulation in serum or plasma [44,45,46,47], whereas others have described decreased expression levels [48,49]. In agreement with our findings, additional studies have found no significant differences between patients with PCa and non-cancer controls [50]. Taken together, the inconsistencies observed across studies, including our own findings, highlight unresolved pre-analytical and analytical challenges that must be addressed before cf-miRNAs can be reliably translated into clinical practice. In 2023, the National Cancer Institute published evidence-based best practice guidelines for cf-miRNA blood collection and processing to help address these standardization gaps, along with comprehensive quality-control panels [39]. Head-to-head methodological comparisons have begun to identify the most robust and reproducible analytical workflows for cf-miRNA quantification; however, consensus regarding optimal protocols remains elusive [51,52].

While altered levels highlight the potential of cf-miRNAs as non-invasive biomarkers, their presence in plasma may also reflect an active mediator role in systemic crosstalk. Emerging evidence, largely pioneered by Lyden and colleagues, demonstrates that tumor-derived molecules, protected from degradation by EVs or protein complexes, such as occurs with miRNAs, can circulate as systemic messengers [53]. These stable circulating factors can be horizontally transferred to recipient cells to modulate the tumor microenvironment and orchestrate pre-metastatic niche formation [54]. Indeed, Zeng et al. provided a landmark of evidence that EV-miR-25-3p induces vascular leakiness and promotes colorectal cancer (CRC) metastasis to the liver and lung in mice [34], raising the possibility that the elevated circulating miR-25-3p observed in our cohort likewise reflects an active oncogenic signaling network rather than passive tumor shedding.

Consistent with this interpretation, our functional assays in the LNCaP cell line demonstrated that increased levels of miR-25-3p significantly enhanced cell proliferation, migration, and invasion, reinforcing its active role in promoting PCa aggressiveness. In addition to our findings in LNCaP cells, this pro-tumorigenic behavior has been documented in various prostate cancer models, such as DU145, 22Rv1 and LNCaP [55], as well as in other solid tumors. Specifically, elevated levels of miR-25-3p have been reported to promote proliferation, migration, and invasion and inhibit apoptosis in cancer cell lines derived from multiple organs, including bladder (T24, 5637) [56,57], breast (MDA-MB-231 and MDA-MB-468) [58], colorectal (HCT116, Caco-2, 293T) [59], and non-small cell lung cancer (A549, H1299) cell lines [12,13]. Although most studies support the role of miR-25-3p as an oncogene, it is important to acknowledge the findings of Zoni et al., who described a context-specific tumor-suppressive function in ALDH^+^ prostate cancer stem cells, where miR-25-3p reduced extravasation by targeting integrins [60].

It is well known that miRNAs can function either as oncogenes or tumor suppressors, depending on the genes they regulate [61]. Given that a single miRNA can exert multiple effects across different biological systems [62,63,64], a functional pattern of miRNA–mRNA regulatory network was analyzed based on miRTarBase to provide insight into the oncogenic behavior observed in the prostate cell line during experimental validation. In the present study, the functional analysis of miR-25-3p involved predicting its target genes and conducting functional enrichment analysis of these targets. Transfection experiments followed by RT-qPCR showed that miR-25-3p can modulate the expression of important genes involved in multiple biological pathways, including epigenetic modifiers (*EZH2*); suppression of metastasis, cell migration, and invasion (*CDH1*); genes related to apoptosis (*BCL2L11*); and cell cycle arrest (*TP53* and *CDKN1C*).

Although the RT-qPCR data only reflect the effect of elevated miR-25-3p abundance on target gene expression rather than direct regulatory binding, our findings support the idea that miR-25-3p acts as an oncogenic miRNA in PCa.

## 4. Materials and Methods

### 4.1. Clinical Samples

From 2016 to 2018, 80 patients were enrolled at Londrina Cancer Hospital (Londrina, Paraná, Brazil). The inclusion criteria for this study were patients with histopathological confirmation of PCa by transrectal needle biopsy. As controls, 40 cancer-free individuals without urinary disease symptoms and PSA levels ≤ 4.0 ng/mL were enrolled at the Consortium Intermunicipal Health of the Middle Paranapanema (CISMEPAR) and Brotherhood of Santa Casa de Londrina (Londrina, PR, Brazil). Medical and pathological reports were obtained, and the demographic, clinical, and histopathological characteristics of patients with PCa and control individuals were analyzed. All participants provided written informed consent and answered a modified questionnaire based on Carrano and Natarajan [65].

Peripheral blood was drawn from patients with no prior cancer treatment and controls during their urology check-up. Whole blood samples were collected using EDTA tubes and were processed within 2 h after collection, following the protocol outlined by Souza et al. 2017 [23]. Plasma was chosen as the biofluid type because the coagulation process during serum preparation can release intracellular miRNAs from blood cells, potentially altering the circulating miRNA profile [38].

### 4.2. miRNA Selection and Target Prediction

Our approach was guided by a systematic effort to address current knowledge gaps and reconcile existing discrepancies in the literature. The miRNAs (miR-25, miR-92a-1, miR-92a-2, and miR-148a) were selected from the remaining 42 candidates based on our previous study [23]. The selection criteria were based on distinct clinical knowledge gaps: miR-92a-1-5p and miR-92a-2-5p have not, to our knowledge, been evaluated in the circulation of patients with PCa; miR-25-3p has only been assessed in serum [36], making plasma validation highly relevant; and miR-148a-3p has shown inconsistent expression patterns across prior studies [44,45,48,49]. The selection criteria were also based on the following parameters: fold change (FC) > 2, nominal *p*-value < 0.001, and false discovery rate (FDR) < 0.001 (Supplementary Table S1). These specific targets ranked 6th, 14th, 23rd, and 28th by FC, respectively, in the TCGA list, while the rankings of the miRNAs selected in the previous study were 1st, 5th, 8th, 39th, 40th, 44th, and 45th [23].

Briefly, using the PCa data of the public dataset, The Cancer Genome Atlas (TCGA), we selected four miRNAs overexpressed in PCa tissue compared to normal prostate tissue. This overexpression was further validated through comparison of normal tissue samples (*n* = 51) and primary tumor samples (*n* = 490) from the TCGA miRNA dataset for the prostate adenocarcinoma cohort, using UALCAN (http://ualcan.path.uab.edu) on 15 December 2024.

Using in silico tools, miRNA target genes were selected based on enrichment analysis using the MicroRNA Enrichment TURned NETwork (MIENTURNET; http://userver.bio.uniroma1.it/apps/mienturnet/ accessed on 15 December 2024) and filtered for experimentally validated interactions from miRTarBase only for strong-evidence interactions between mRNA and miRNA [66]. This analysis provided 26 candidates genes, of which we chose 6 (*TP53*, *MDM2*, *BCL2L11*, *CDH1*, *CDKN1C* and *EZH2*) that fitted the following criteria: (i) the availability of validated primers in our laboratory’s primer bank; (ii) biological relevance to PCa and relevant cancer-associated pathways; and (iii) a minimum threshold of one miRNA–target interaction.

### 4.3. Cell-Free RNA Isolation and Analysis of miRNA Expression with RT-qPCR

Extraction of total cell-free RNAs was performed using the miRNeasy Serum/Plasma Kit (217184, Qiagen, Hilden, Germany) with minor modifications to the manufacturer’s protocol [23,67]. Samples were quantified using a NanoDrop 2000 spectrophotometer (Thermo Fisher Scientific, Waltham, MA, USA). For PCa patients and controls, the expression patterns of miRNAs (hsa-miR-25-3p [000403], hsa-miR-92a-1-5p [002137], hsa-miR-92a-2-5p [002138] and hsa-miR-148a-3p [000470]) were performed using 5 ng of total RNA and a TaqMan miRNA Reverse Transcription kit (PN4427975, Applied Biosystems, Foster City, CA, USA), following the manufacturer’s instructions. A 1:4 dilution of miRNA cDNA was used as input for the qPCR reaction. The qPCR reaction was performed using 5.5 μL of TaqMan 2X Universal PCR Master Mix (4440038, Applied Biosystems, Foster City, CA, USA), 0.45 μL of miRNA-specific TaqMan Probe (PN4427975, Applied Biosystems, Foster City, CA, USA), and 7 μL of diluted miRNA cDNA on the Techne Quantum™ Real-Time PCR Cycler System (Techne, Stone, Staffordshire, UK) at 50 °C for 2 minute (min), 95 °C for 10 min and 50 cycles at 95 °C for 15 s and 60 °C for 1 min. U6 snRNA [001973] and RNU48 [001006], which demonstrated reproducible Ct values across all samples, were utilized as endogenous controls for normalization. A pooled sample consisting of 2 µL of total RNA from each sample across the three groups was used as a calibrator in all RT-qPCR plates to ensure experimental consistency.

### 4.4. Cell Culture and Cell Transfection Conditions

The prostate cancer androgen-dependent cell line (Lymph Node Carcinoma of the Prostate—LNCaP) was obtained from the American Type Culture Collection catalog number: CRL-1740, (ATCC, Manassas, VA, USA) and cultured in RPMI 1640 Medium (11875093, Gibco, Grand Island, NY, USA) containing 15% fetal bovine serum (12483-020, Gibco, Burlington, ON, Canada) and 1% gentamicin reagent solution (15710-064, Gibco, Shanghai, China). The cells were cultured in a humidified incubator at 37 °C and 5% CO_2_.

The oligonucleotides miR-25-3p mimic (4464066, #MC10584, Ambion, Austin, TX, USA) and mirVana™ miRNA Mimic, negative control (NC) #1 (4464058, Ambion, Austin, TX, USA) were transfected at a final concentration of 10 nM, while the anti-miR-25-3p mimic (4464066, #MH10584, Ambion, Austin, TX, USA) was transfected at a final concentration of 50 nM. All transfections were performed using Lipofectamine 2000 (11668-027, Invitrogen, Carlsbad, CA, USA) following the manufacturer’s instructions. LNCaP cells were cultured in transfection medium for 24, 48, and 72 h in an incubator at 37 °C and 5% CO_2_ and were subsequently subjected to gene expression and functional assays. The quantification of miR-25-3p abundance in cell culture was conducted according to the protocol described in Section 2.4.

### 4.5. Gene Expression Analysis (RT-qPCR)

For analysis of the gene expression of the target genes of hsa-miR-25-3p (*BCL2l1, CDH1, CDKN1C, EZH2, MDM2,* and *TP53*), LNCaP cells (5 × 10^3^) were seeded in triplicate in 96-well plates, transfected and maintained with the mimic and controls for a period of incubation of 48 h. The total RNA was isolated from cells using QIAzol Lysis Reagent (79306, Qiagen, Hilden, Germany) and quantified by spectrophotometry using NanoDrop 2000 (Thermo Fisher Scientific, Waltham, MA, USA).

Briefly, first-strand cDNA synthesis was performed with the Transcriptor First Strand cDNA Synthesis Kit (04896866001, Roche Applied Science, Penzberg, Germany) using 1 μg of total RNA according to the manufacturer’s instructions on a Mastercycler Nexus Gradient PCR machine (Eppendorf, Hamburg, Germany), under the following conditions: 25 °C for 10 min, 30 °C for 30 min, and the reaction terminated at 85 °C for 5 min. The resulting cDNA was diluted in 120 μL nuclease-free water. The specific primer sets used in RT-qPCR are listed in Supplementary Table S2. Reactions were performed using SensiFAST™ SYBR^®^ No-ROX Kit (BIO-98005, Bioline, London, UK) according to the protocol in a CFX Connect Real-Time PCR Detection System (Bio-Rad, Hercules, CA, USA). The housekeeping β-Actin gene was used as an endogenous control. 

### 4.6. Cell Proliferation Assay

Proliferation assays were performed in 96-well dishes with 2 × 10^3^ LNCaP cells seeded per well, with 10 replicates per condition. One field was imaged per well under 4× magnification every 24 h for 96 h. Proliferation was measured as a function of cell confluence by live-cell microscopy. Data were analyzed using the IncuCyte^®^ S3 software (version 2019, Essen Biosciences (Sartorius), Ann Arbor, MI, USA), which quantified cell surface area coverage as confluence values.

### 4.7. Colony Formation Assay

The colony-forming assay measured the clonogenicity of cells. Transfected cells were detached, and 2500 cells per well were plated in a 6-well plate with 10% fetal bovine serum (FBS) for 14 days to form colonies. The cells were then washed twice with PBS, immediately fixed with 1 mL of methanol at room temperature for 15 min, and subsequently stained with a 5% Giemsa solution (Solarbio, Beijing, China) for 30 min at room temperature. After removing the excess stain and drying, the incorporated dye was solubilized using 10% acetic acid. Following this, we measured the absorbance of the clonogenic assay at 590 nm using a microplate reader (FLUOstar Omega; BMG Labtech, Ortenberg, Germany).

### 4.8. Cell Migration and Invasion Assay

The migration and invasion of PCa cells were analyzed in 24-well transwell chambers with 8 μm pore size polycarbonate membranes (662638, Greiner Bio-One, Frickenhausen, Germany). For the migration assay, a total of 5 × 10^4^ cells suspended in 200 μL of FBS-free medium were seeded into the upper chamber. The lower chamber was filled with 500 μL of medium supplemented with 10% FBS to serve as a chemoattractant.

For invasion assays, a Matrigel-coated membrane (354480, BD BioCoat™, Bedford, MA, USA) was used under the same conditions described above. After 48 h of incubation, cells remaining on the upper surface of the membrane were removed with cotton swabs, while cells on the lower surface were fixed in methanol for 15 min and stained with 5% Giemsa solution (Solarbio, Beijing, China) for 30 min. Cells were counted in all four randomly selected fields using a light microscope (EVOS XL Core, Thermo Fisher Scientific, Waltham, MA, USA) at 10× magnification.

### 4.9. Statistical Analysis

Descriptive analysis of demographic and clinical data was carried out using IBM SPSS Statistics 22.0 (IBM Corp., Armonk, NY, USA). The relative expression of miRNAs and mRNAs was calculated using the 2^−ΔΔCt^ method [67,68]. Data were reported as mean values ± standard deviation (SD) of at least three technical replicates. Data distribution and homogeneity of variances were evaluated using the Shapiro–Wilk and Levene’s tests, respectively. Subsequently, either the unpaired Student’s *t*-test or one-way ANOVA was applied to compare means between groups. Diagnostic performance was evaluated using receiver operating characteristic (ROC) curve analysis to determine sensitivity, specificity, and the area under the curve (AUC) alongside 95% confidence intervals (CIs). Statistical analyses were performed using GraphPad Prism (Version 10.4.2 (534), GraphPad Software, Boston, MA, USA). A *p*-value < 0.05 was considered statistically significant.

## 5. Conclusions

LB offers a more comprehensive view of the tumor landscape than static tissue biopsies. Our study identifies miR-25-3p as a potential minimally invasive biomarker for PCa, as its plasma abundance is significantly elevated in patients and correlates with higher ISUP grades. Functional validation in LNCaP cells confirms that miR-25-3p promotes cell proliferation and invasion, indicating that it acts as an active oncogenic mediator rather than a passive byproduct of tumor burden. Notably, miR-92a-1-5p and miR-92a-2-5p were evaluated for the first time in patients with PCa but showed no potential as minimally invasive biomarkers. Additionally, our findings for miR-25-3p and miR-148a-3p contrast with the previously published literature, reflecting the complex biology of cf-miRNAs as well as the impact of methodological and ethnic variability. These findings underscore the urgent need for standardized protocols and multicenter studies to successfully translate these data into clinical practice.

## Figures and Tables

**Table 1:** Demographic and clinical characteristics of patients with prostate cancer and controls.

Characteristics		PCa	Controls	*p*-Value
		*n* (%)	*n* (%)	
Age	≤64	31 (38.8)	21 (52.5)	
	≥65	49 (61.3)	19 (47.5)	0.154
Ancestry	Afro-descendant	17 (21.3)	7 (17.5)	
	Euro-descendant	63 (78.8)	33 (82.5)	0.631
Family history	No	32 (40.0)	23 (57.5)	
of cancer	Yes	34 (42.5)	15 (37.5)	0.168
	Yes, prostate	14 (17.5)	2 (5.0)	0.044 *
Vasectomy	No	78 (97.5)	40 (100.0)	
	Yes	2 (2.5)	0 (0.0)	0.999
Occupational	No	20 (25.0)	11 (27.5)	
exposure	Yes, agrochemicals	42 (52.5)	19 (47.5)	0.670
	Yes, others	18 (22.5)	10 (25.0)	0.985
PSA (ng/mL)	≤4.0	8 (10.0)	40 (100.0)	
	4.1 to 10.0	32 (40.0)	0 (0.0)	
	>10.0	40 (50.0)	0 (0.0)	
Treatment	Active surveillance	33 (41.3)	n.a.	
	Prostatectomy	46 (57.5)	n.a.	
	TURP	1 (1.3)	n.a.	
ISUP grade	1 (Gleason Score = 6)	14 (17.5)	n.a.	
(Radical prostatectomy)	2 (Gleason Score = 3 + 4)	25 (31.3)	n.a.	
	3 (Gleason Score = 4 + 3)	6 (7.5)	n.a.	
	4 (Gleason Score = 8)	2 (2.5)	n.a.	
	5 (Gleason Score = 9–10)	0 (0.0)	n.a.	
	NS	33 (41.2)	n.a.	
Tumor laterality	No	29 (36.3)	n.a.	
	Yes	17 (21.3)	n.a.	
	NS	34 (42.4)	n.a.	
Seminal vesicle	No	44 (55.0)	n.a.	
invasion	Yes	3 (3.8)	n.a.	
	NS	33 (41.2)	n.a.	
Perineural invasion	No	43 (53.8)	n.a.	
	Yes	3 (3.8)	n.a.	
	NS	34 (42.4)	n.a.	
Lymph node	No	45 (56.3)	n.a.	
metastasis	Yes	1 (1.3)	n.a.	
	NS	34 (42.5)	n.a.	
Bone metastasis	No	65 (81.3)	n.a.	
	Yes	11 (13.7)	n.a.	
	Not evaluated	4 (5.0)	n.a.	

PCa: prostate cancer; Yes: presence; No: absence; TURP: transurethral resection of the prostate; NS: not surgery; PSA: prostate-specific antigen; ISUP: International Society of Urological Pathology; n.a.: not applicable; * *p* < 0.05.

## Data Availability

The datasets analyzed during the present study are available from the corresponding author upon reasonable request.

## Supplementary Materials

The following supporting information can be downloaded at: https://www.mdpi.com/article/10.3390/ncrna12050033/s1, Supplementary Figure S1. Effects of miR-25-3p inhibition in LNCaP cells. (**a**) Relative expression (log2) of miR-25-3p with a negative control (NC) or anti-miR-25-3p; (**b**) cell proliferation evaluated by confluence percentage over 72 h; (**c**) clonogenic assay measuring colony formation (absorbance at 590 nm), with representative plate images; (**d**) migration assay quantifying cell number per field, with representative micrographs; (**e**) invasion assay quantifying cell number per field, with representative micrographs. NC: negative control. Supplementary Table S1. Differentially expressed miRNA in the in silico analysis using TCGA. Supplementary Table S2. Primer sequences used for RT-qPCR. Supplementary Table S3. Candidate target genes of miR-25-3p based on miRTarBase.
