# Supplementary material for: Circulating Cell-Free miR-25-3p Is Upregulated in Brazilian Patients with Prostate Cancer and Contributes to Tumor Aggressiveness
Source: Noncoding RNA. 2026 Aug 30;12(5):33. doi: 10.3390/ncrna12050033 (PMC13600208; doi:10.3390/ncrna12050033)
Supplement: Supplementary file 1 [file ncrna-12-00033-s001.zip › Supplementary Table S1 - Nobrega et al..pdf]

Table S1. Differentially expressed miRNA in the in silico analysis using TCGA.

microRNA ID	Parametric P value	FDR	R-CaP	R-CTL	Fold-Change
hsa-miR-92a-2	< 1e-0	< 1e-07	26858,16	12931,03	2,08
hsa-miR-25	< 1e-07	< 1e-07	26109,99	11659,61	2,24
hsa-miR-148a	< 1e-07	< 1e-07	236706,14	85496,45	2,77
hsa-miR-92a-1	< 1e-0	< 1e-07	2827,86	698,8	4,05

hsa: Human Species; **FDR:** false discovery rate; **R-CaP:** geometric mean of intensities in class tumor; **R-CTL:** geometric mean of intensities in class normal.
