# Supplementary material for: Circulating Cell-Free miR-25-3p Is Upregulated in Brazilian Patients with Prostate Cancer and Contributes to Tumor Aggressiveness
Source: Noncoding RNA. 2026 Aug 30;12(5):33. doi: 10.3390/ncrna12050033 (PMC13600208; doi:10.3390/ncrna12050033)
Supplement: Supplementary file 1 [file ncrna-12-00033-s001.zip › Supplementary Table S2 - Nobrega et al..pdf]

Table S2. Primer sequences used for RT-qPCR.

Gene	Primer sequence (5′-3′)
B-ACTIN	Forward: ACCTTCTACAATGAGCTGCG
	Reverse: CCTGGATAGCAACGTACATGG
BCL2L11	Forward: CCGCCCTTATGATGAAGTGT
	Reverse: AAAGCCTGGAGTCAGCAAAA
CDH1	Forward: CTCAAAATCCTCCCTGTCCA
	Reverse: TGGAGAGACACTGCCAACTG
CDKN1C	Forward: ATGCCCATCTAGCTTGCAGT
	Reverse: GGTACAGACGGCTCAGGAAC
EZH2	Forward: TTCATGCAACACCCAACACT
	Reverse: CTCCCTCCAAATGCTGGTAA
MDM2	Forward: CCGAATAAGGTTTGCCTGAA
	Reverse: CAAATTGCAAAAGGCACTGA
TP53	Forward: GGCCCACTTCACCGTACTAA
	Reverse: GTGGTTTCAAGGCCAGATGT
