# Supplementary material for: Circulating Cell-Free miR-25-3p Is Upregulated in Brazilian Patients with Prostate Cancer and Contributes to Tumor Aggressiveness
Source: Noncoding RNA. 2026 Aug 30;12(5):33. doi: 10.3390/ncrna12050033 (PMC13600208; doi:10.3390/ncrna12050033)
Supplement: Supplementary file 1 [file ncrna-12-00033-s001.zip › Supplementary Table S3 - Nobrega et al..pdf]

Table S3. Candidate target genes of has-miR-25-3p based on miRTarBase.

Gene Symbol	p-value	FDR	Number of interactions	microRNA
CDKN1C	0,003078107	0,023797902	1	hsa-miR-25-3p
CDH1	0,008464794	0,031406019	1	hsa-miR-25-3p
EZH2	0,016929588	0,036088587	1	hsa-miR-25-3p
BCL2L11	0,046941131	0,05076306	1	hsa-miR-25-3p
TP53	0,050019238	0,053094191	1	hsa-miR-25-3p
MDM2	0,078106964	0,079176923	1	hsa-miR-25-3p

hsa: Human Species; **FDR:** false discovery rate.
